# Case-Based Study: From Prediabetes to Complications—Opportunities for Prevention

**DOI:** 10.1371/journal.pmed.0020040

**Published:** 2005-02-22

**Authors:** Jonathan Rappaport, Vivian Fonseca

## Abstract

A 31-year-old man presents with central obesity, hypertension, and abnormal lipids. How would you manage this patient?

## DESCRIPTION of CASE

A 31-year-old white male with no significant past medical history is referred by his workplace to a primary care physician for an elevated blood pressure (BP). He presents to the clinic with no complaints. His mother and grandmother both have diabetes, and his father has hypertension. He has had a 15-pound (lb) weight gain over the last year and has become more sedentary.

His BP is 142/90 mm Hg, pulse is 88 beats per minute (bpm), weight is 209 lb, and height is 5′ 11″. On examination he displays moderate central obesity, but otherwise the examination is normal. His fasting cholesterol is 228 mg/dl (to convert milligrams per deciliter of cholesterol [total, HDL or LDL] to micromoles per liter, divide by 39), low-density lipoprotein (LDL) is 166 mg/dl, high-density lipoprotein (HDL) is 32 mg/dl, triglycerides (TG) are 223 mg/dl (to convert mg/dl of triglycerides to mmol/l, divide by 89), and fasting glucose is 114 mg/dl (to convert mg/dl of glucose to mmol/l, divide by 18).

### What Is the diagnosis?

This patient meets the diagnostic criteria for the metabolic syndrome as defined by the National Cholesterol Education Program Adult Treatment Panel III guidelines [1]. Any three or more of the criteria make this diagnosis (see Table 1). Intensive lifestyle modifications such as exercise and weight loss should be made to improve cholesterol, blood pressure, and other cardiovascular disease (CVD) risk factors [2]. It may be timely to address the prevention of diabetes in patients with metabolic syndrome since these patients are at high risk for development of type 2 diabetes. Lifestyle changes delay the onset or prevent the incidence of type 2 diabetes in patients with glucose intolerance, a key feature of metabolic syndrome [3]. The patient is started on an exercise and weight loss program, sent for nutritional counseling, and scheduled for a return clinic appointment for three months later.

**Table 1:**
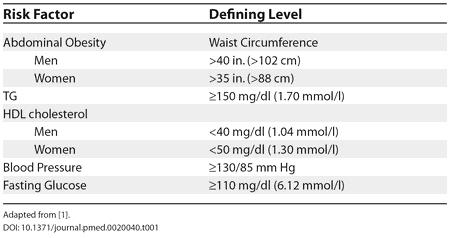
National Cholesterol Education Program Clinical Identification of the Metabolic Syndrome

Adapted from [1]

### Two Years Later

The patient returns to the clinic two years later. He presents with complaints of increasing frequency of urination and episodes of blurry vision. He has nocturia and has lost 5 lb in the last week. Otherwise, his review of systems is unremarkable. His blood pressure is 146/88 mm Hg, pulse 80 bpm, and weight 216 lb. His fundoscopic examination is normal. He continues to have moderate central obesity. Current medications are a thiazide diuretic, 12.5 mg once daily (QD), started one year prior. A non-fasting blood sugar is 267 mg/dl.

#### Can a diagnosis be made?

There are three criteria for the diagnosis of type 2 diabetes as defined by the American Diabetes Association (ADA), of which any one is sufficient to make the diagnosis (see Box 1). This patient meets the criteria for type 2 diabetes. He does not need to have a fasting blood sugar done because a random glucose greater than 200 mg/dl with symptoms of diabetes meets the first criterion. Failing to comply with lifestyle modification, his weight has increased 7 lb in two years and likely contributes to his development of diabetes. Of note, his recent weight loss is presumably due to overt hyperglycemia and glycosuria, further underestimating his true weight increase.

**Box 1. ADA Diagnostic Criteria for Type 2 Diabetes**

1. Random plasma glucose ≥200 mg/dl (11.1 mmol/l) and symptoms **or**
2. Fasting plasma glucose =126 mg/dl^a^ (6.99 mmol/l) **or**
3. Two-hour plasma glucose =200 mg/dl^a^ (11.1 mmol/l) in oral glucose tolerance test.

^a^In the absence of symptoms, these criteria should be confirmed by repeat testing on a different day.

Source: [28].

His additional investigations are as follows: fasting glucose, 215 mg/dl; hemoglobin A1c (HbA_1c_), 8.6%; and urine albumin-to-creatinine ratio, 2.0 mg/mmol (normal is <2.5 mg/mmol in men and <3.5 mg/mmol in women). LDL is 176 mg/dl, HDL 32 mg/dl, and TG 292 mg/dl. His electrocardiogram is normal.

### What are the next steps in management at this time?

Diabetes management should involve a multifaceted, goal-directed approach, which includes dietary modifications, diabetes education, assessment of blood sugar readings, and pharmacotherapy. The ADA recommends glycemic and other CVD risk factor goals (see Table 2), in addition to foot evaluation and screening for nephropathy and retinopathy, for all adults with diabetes [4]. The patient is started on metformin, 500 mg twice daily (BID) with meals. Therapy with metformin appears to decrease the risk of diabetes-related endpoints, including a reduction in cardiovascular events independent of glycemic control. There is also less weight gain and fewer hypoglycemic attacks than with insulin and sulphonylureas. Therefore, metformin may be an effective first-line pharmacotherapy of choice in these patients [5]. There are several oral hypoglycemic agents (i.e., sulfonylureas, metformin, acarbose, and thiazolidinediones) that are effective monotherapy for reducing hyperglycemia.

**Table 2:**
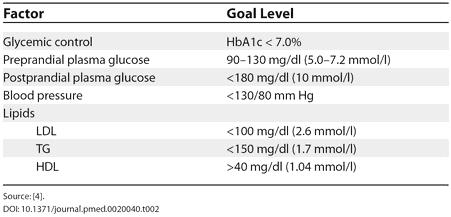
ADA Summary of Goals in Adult Patients with Diabetes

Source: [4]

The patient is also started on low-dose aspirin, indicated for primary prevention of macrovascular disease in people with diabetes who have any risk factors for CVD [4], and a cholesterol-lowering agent, a statin, for his increased LDL cholesterol [6]. He is given a glucose meter, is scheduled to have diabetes education classes and diabetes nutritional counseling for a 1,800-calorie ADA diet, and is instructed to record his pre-meal blood sugars. Smoking cessation is another important aspect of diabetes management to address. He returns in three months for follow-up and has an HbA1c of 7.3%, at which time no additional therapy is started.

### Three Years Later

The patient is now 37 years old and returns for a follow-up appointment. He states that he has felt “pins and needles” in his feet and fingertips. He has had difficulty with maintaining erections but has a normal libido. Blood sugars are 160–190 mg/dl in the mornings and 200–240 mg/dl in the evenings, and the patient reports no hypoglycemic events. He has diminished sensation to vibration over his right great toe and left toes and heel with intact monofilament sensation. The remainder of his examination is unchanged. His medications are metformin at 1 g BID, a thiazide diuretic at 25 mg QD, a statin QD, and an aspirin QD. He is 215 lb, BP is 142/86 mm Hg, and pulse is 76 bpm. Recent laboratory tests produced the following results: a HbA1c of 8.1%, a fasting glucose of 212 mg/dl, and normal electrolytes, creatinine, and liver enzymes. Fasting lipids are LDL 144 mg/dl, HDL 33 mg/dl, and TG 209 mg/dl.

#### What additional diagnostic tests would be helpful at this time, and why?

A spot urine albumin-to-creatinine ratio is 7.6 mg/mmol. This measurement technique is preferred because it has lower rates of false-positive and false-negative results than a spot urine microalbumin. Persistent microalbuminuria should be confirmed on two or three subsequent readings within a six-month period to rule out false-positive results. The elevated ratio of microalbumin in the urine signifies early nephropathy because microalbuminuria has been shown to progress to macroalbuminuria and eventual nephropathy in type 1 and type 2 diabetes. Any degree of albuminuria is a risk factor for cardiovascular events in individuals with or without diabetes; the risk increases with the level of absolute microalbuminuria [7]. Therefore, screening for microalbuminuria should be done annually in all people with type 1 and type 2 diabetes [8].

**Figure 1:**
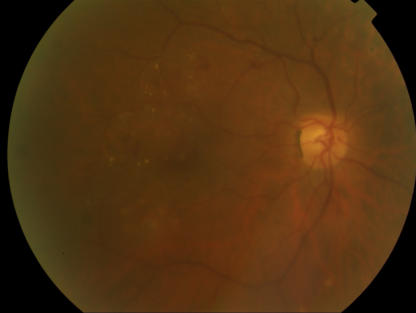
Very Mild Diabetic Retinopathy

**Figure 2 pmed-0020040-g002:**
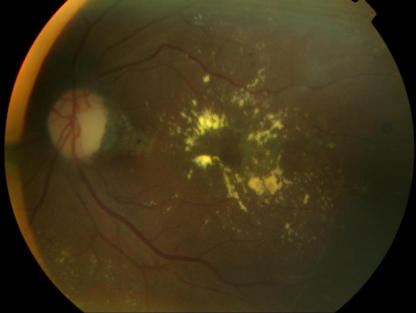
Non-Proliferative Diabetic Retinopathy Showing Several Exudates around the Macula

**Figure 3 pmed-0020040-g003:**
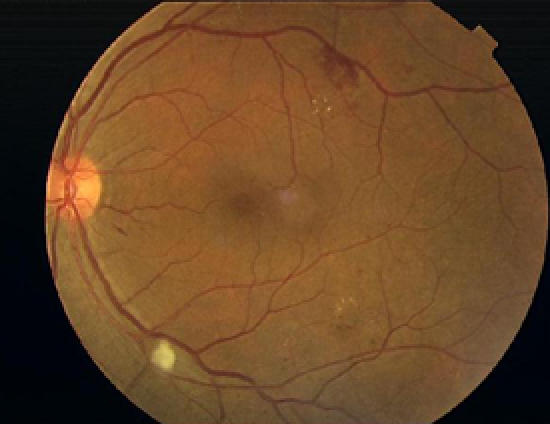
Non-Proliferative Diabetic Retinopathy Showing Macular Edema, a Cotton-Wool Spot below the Optic Disk, and a Few Hemorrhages and Exudates

Annual screening for diabetic retinopathy should be performed in all people with diabetes after an initial evaluation and reassessed more frequently if retinopathy is diagnosed. This patient remains free of retinopathy, but a significant number of patients with type 2 diabetes have retinopathy at the onset of diagnosis owing to the insidious nature of type 2 diabetes and the failure to diagnose type 2 diabetes early. Tight glycemic control can slow the progression of diabetic retinopathy (Figures 1–3 [Fig pmed-0020040-g002] [Fig pmed-0020040-g003]) [9] and help prevent development of proliferative diabetic retinopathy.

#### What additional pharmacotherapy should be started at this time?

The patient has developed neuropathy and erectile dysfunction, both of which are complications of diabetes. He continues to have suboptimal glycemic control; therefore, additional therapy in the form of combinations is appropriate. The patient is started on a thiazolidinedione (TZD) QD. With continued elevated systolic BP >130 mm Hg and diastolic BP >80 mm Hg, an angiotensin-converting enzyme inhibitor (ACE-I) is started. An ACE-I at this time is appropriate for BP control and has the additional preventative effects of reducing progression to nephropathy and CVD events [10,11]. In addition, continued strict BP control is as effective as tight glycemic control in preventing macrovasular disease in diabetic patients and slowing the progression of diabetic nephropathy and retinopathy [12]. Erectile dysfunction is a complication associated with diabetes and can be an early sign of neuropathy and vascular disease, therefore a phosphodiesterase-5 enzyme inhibitor is an appropriate choice for patients not on vasodilators or with a history of significant CVD. The statin dose is increased to achieve a goal LDL of ≤100 mg/dl. Diabetic neuropathy is a significant cause of morbidity in diabetes, and its progression correlates directly with glycemic control. Tighter glucose control and proper foot care are effective. It is important to continue emphasis on dietary, exercise, and lifestyle modifications in addition to pharmacotherapy.

### Five Years Later

The patient returns to clinic today after spending the last three years overseas and has not seen a physician in two years. He complains of fatigue, occasional blurry vision, awakening three to four times at night to urinate, and diarrhea at least once a week. He says that he has been compliant with his diabetes medications but has gained 15 lb in the last six months. His medications include metformin at 1 g BID, a TZD BID, and an ACE-I QD. His blood sugar is 289 mg/dl (fasting), BP is 130/90 mm Hg, pulse is 88 bpm, and weight is 221 lb. There are no foot sores or ulcers, but he has diminished sensation to monofilament on the plantar surfaces of both feet. The remainder of his examination is unchanged, including normal fundoscopy. His HbA1c is 9.6%, LDL is 143 mg/dl, and spot urine albumin-to-creatinine ratio is 15 mg/mmol. His creatinine and liver enzymes are normal. His pre-meal blood sugars average 210–250 mg/dl.

#### What is the next most appropriate step in his medical management?

He continues to have an elevated HbA1c, worsening neuropathy, and weight gain, which prompt a more effective treatment strategy. There are several options for pharmacotherapy available to choose from at this point. The patient could begin a third oral agent after maximizing the doses of metformin and TZD, or he could begin insulin injections with or without additional oral agents. Because of the significant cost associated with three oral medications and his need for further glycemic control, insulin would be an appropriate choice at this time. However, he should be advised of the side effect of additional weight gain when beginning insulin therapy.

## DISCUSSION

This case presentation illustrates an otherwise healthy appearing patient who is found to have the metabolic syndrome and despite evidence-based management develops type 2 diabetes. This patient likely represents the natural history of type 2 diabetes in most patients. Mild hypertension is often the only presenting sign of metabolic syndrome and prediabetes, allowing an opportunity for prevention of type 2 diabetes.

There is an association between metabolic syndrome and the development of CVD and type 2 diabetes [13]. This syndrome is characterized not only by the criteria given in Table 1, but also by a state of compensatory hyperinsulinemia [14]. However, a diagnosis of metabolic syndrome alone does not imply diabetes, as patients with metabolic syndrome can have a fasting plasma glucose less than 110 mg/dl. It is the body's ability to maintain glucose utilization and suppress endogenous glucose production in the setting of this compensatory hyperinsulinemia that separates metabolic syndrome from diabetes. The effect of this hyperinsulinemic state in metabolic syndrome is also believed to be involved in excess pro-inflammatory and pro-thrombotic markers associated with the development of diabetes and CVD [15]. These patients develop diabetes when tissues of the body fail to utilize glucose appropriately owing to increased resistance to insulin and concomitant beta-cell dysfunction of the pancreas [16].

Metformin is in the class of biguanides and works by decreasing hepatic glucose output and increasing insulin action in tissues. Metformin has been suggested to help prevent the onset of diabetes but is less effective than diet and lifestyle changes [3]. Other medications shown to possibly delay or prevent the onset of type 2 diabetes are ACE-I and angiotensin II receptor blockers [17,18]. Patients treated with diuretics can progress to type 2 diabetes even though thiazide diuretics are proven effective in treating hypertension [19,20].

Intensive therapy in patients with type 2 diabetes results in a decreased risk of microvascular complications; therefore, it is appropriate to use combinations of medications in patients with suboptimal glycemia [21]. The class of TZDs works to lower plasma glucose levels by increasing insulin sensitivity in muscle and liver [22]. TZDs lower mean HbA1c modestly when added to metformin as compared to metformin alone [23]. Side effects include weight gain and water retention, and patients with a history of New York Heart Association class III or IV heart failure should not use TZDs [24,25].

**Figure 4:**
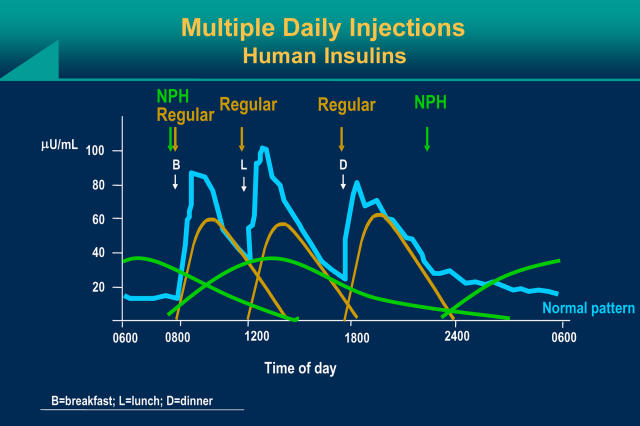
Using a Short-Acting (Regular) Insulin before Meals Provides Insulin Action That Closely Approximates Normal Insulin Secretion

The pathophysiology of type 2 diabetes involves, in part, a “relative” deficiency of insulin. Although a state of endogenous hyperinsulinemia occurs, the degree of tissue resistance causes a total decrease in “effective” endogenous insulin. Progression of disease is also attributed to worsening beta-cell dysfunction and decreased release of insulin [26]. Insulin is used in a variety of combinations and is individualized to patient lifestyle. A frequent starting dose consists of a long- or intermediate-acting insulin, such as NPH insulin, divided into morning and evening doses, or insulin glargine given QD, usually at bedtime. The patient whose case is described here was started on NPH at bedtime, which decreases overnight hepatic glucose production such that the patient begins the morning with near-normal glycemia for daytime oral therapy. There may be times when a post-meal surge in glucose requires extra insulin in addition to the intermediate-acting NPH. In such a case, using a short-acting (regular) insulin before meals provides insulin action that closely approximates normal insulin secretion (Figure 4). The rapid-acting lispro and aspart insulins have an even shorter half-life and quicker onset of action than regular insulin. Common empirical initiation doses range from 0.4–1.2 units of insulin per kilogram per 24 hours. Patients should be advised of hypoglycemia and weight gain as the main side effects of insulin therapy. Insulin and insulin-sensitizer combinations significantly improve hyperglycemia; however, there is an increased incidence of heart failure reported with this combination, prompting close monitoring of patients for signs and symptoms of heart failure [27].

In summary, diabetes prevention and management is an important goal in practice. The morbidity and mortality from diabetes is a significant burden to health care, emphasizing the need for effective prevention and control of diabetes in improving outcomes.

**Editorial Note**

The management of the patient in this Learning Forum article is in keeping with two national guidelines—those of the United States National Cholesterol Education Program and the ADA. Both peer reviewers pointed out that clinicians in other countries would follow their own national or regional guidelines. For example, the guidelines for the management of type 2 diabetes published by the United Kingdom's National Institute for Clinical Excellence differ in key ways from the ADA guidelines. We as editors debated whether to insist that the authors include guidance from other parts of the world. We decided that as an international journal we should reflect global variations in practice and allow authors to discuss how patients would be optimally managed in their own countries. There is much we can learn from different approaches to clinical practice worldwide.—The *PLoS Medicine* Editors

**Useful Links**

National Institute for Clinical Excellence Clinical Guidelines for Type 2 Diabetes: www.nice.org.uk/pdf/NICE_full_blood_glucose.pdf

International Diabetes Federation (European Region) Desktop Guide to Type 2 Diabetes: www.staff.ncl.ac.uk/philip.home/t2dg1999.htm

**Key Learning Points**

- The natural history of diabetes suggests that it is a progressive disease, and therapy may need to be frequently changed or augmented over time.
- The diagnosis of the metabolic syndrome should alert primary care physicians to prescribe intensive lifestyle modifications for prevention of diabetes.
- Strict BP, lipid, and weight control is just as essential as strict glycemic control in preventing CVD in patients with diabetes.
- Metformin can reduce the risk of CVD in obese patients with diabetes independent of glycemic control.
- The decision of combination oral therapy with or without insulin should be individualized to optimize glycemic control and reduce micro- and macrovascular complications.

## Abbreviations

ACE-I: angiotensin-converting enzyme inhibitor
ADA: American Diabetes Association
BID: twice daily
BP: blood pressure
bpm: beats per minute
CVD: cardiovascular disease
HbA1c: hemoglobin A1c
(HDL: high-density lipoprotein
lb: pound
LDL: low-density lipoprotein
QD: once daily
TG: trigycerides
TZD: thiazolidinedione
